# Considerations for preparing a randomized population health intervention trial: lessons from a South African–Canadian partnership to improve the health of health workers

**DOI:** 10.3402/gha.v7.23594

**Published:** 2014-05-02

**Authors:** Annalee Yassi, Lyndsay Michelle O’Hara, Michelle C. Engelbrecht, Kerry Uebel, Letshego Elizabeth Nophale, Elizabeth Ann Bryce, Jane A Buxton, Jacob Siegel, Jerry Malcolm Spiegel

**Affiliations:** 1 School of Population and Public Health, University of British Columbia, Vancouver, Canada; 2 Centre for Health Systems Research & Development, University of the Free State, Bloemfontein, South Africa; 3 Provincial Occupational Health Unit, Free State Department of Health, University of the Free State, Bloemfontein, South Africa; 4 Vancouver Coastal Health, Vancouver, Canada

**Keywords:** cluster-randomized controlled trials, population health, pre-trial considerations, receptor capacity, feasibility studies, pilot testing, building relationships, ethical issues, iterative process

## Abstract

**Background:**

Community-based cluster-randomized controlled trials (RCTs) are increasingly being conducted to address pressing global health concerns. Preparations for clinical trials are well-described, as are the steps for multi-component health service trials. However, guidance is lacking for addressing the ethical and logistic challenges in (cluster) RCTs of population health interventions in low- and middle-income countries.

**Objective:**

We aimed to identify the factors that population health researchers must explicitly consider when planning RCTs within North–South partnerships.

**Design:**

We reviewed our experiences and identified key ethical and logistic issues encountered during the pre-trial phase of a recently implemented RCT. This trial aimed to improve tuberculosis (TB) and Human Immunodeficiency Virus (HIV) prevention and care for health workers by enhancing workplace assessment capability, addressing concerns about confidentiality and stigma, and providing onsite counseling, testing, and treatment. An iterative framework was used to synthesize this analysis with lessons taken from other studies.

**Results:**

The checklist of critical factors was grouped into eight categories: 1) Building trust and shared ownership; 2) Conducting feasibility studies throughout the process; 3) Building capacity; 4) Creating an appropriate information system; 5) Conducting pilot studies; 6) Securing stakeholder support, with a view to scale-up; 7) Continuously refining methodological rigor; and 8) Explicitly addressing all ethical issues both at the start and continuously as they arise.

**Conclusion:**

Researchers should allow for the significant investment of time and resources required for successful implementation of population health RCTs within North–South collaborations, recognize the iterative nature of the process, and be prepared to revise protocols as challenges emerge.

Biomedical and clinical research seeks to understand disease dynamics and to inform the development of therapeutic and diagnostic modalities, whereas population health research primarily aims to develop and implement solutions to reduce the burden of disease, minimize health inequities, and inform practice to improve the overall health of populations (1, 2). Randomized controlled trials (RCTs) have been encouraged in population health, in part to augment the internal validity of studies (3, 4) and, indeed, excellent cluster RCTs are increasingly being conducted (e.g. 5).

Steps necessary to initiate *clinical* RCTs are well-described (3, 4); the Medical Research Council (MRC) in the United Kingdom has published a framework for developing and evaluating RCTs for *complex interventions* defined as ‘interventions with several interacting components’ (6, 7); others have proposed principles for effective research *collaborations* (8). However, while the additional challenges posed by North–South collaborations have been extensively highlighted (9, 10), guidance for addressing these in *population health* RCTs have not been systematically analyzed (11, 12) let alone the implications for addressing such dilemmas in low- and middle-income countries (LMICs).

We, therefore, analyzed our own seven years of experience in developing and launching one such RCT, and drew on evidence from other similar studies to develop a theoretical understanding of the process, and to identify challenges and lessons learned. Every stage of the development–evaluation–implementation process of a complex intervention is important and, therefore, takes considerable time (6). Unlike the reductionist approach of the clinical RCT, complex population health interventions necessitate a realist approach, which is context dependent and discerns what works for whom, in what circumstances, and how (13).

This article draws upon our collaborative Canadian–South African research program to improve the health of health workers in Free State, South Africa, especially with respect to morbidity and mortality associated with tuberculosis (TB) and the Human Immunodeficiency Virus (HIV). The HIV epidemic puts enormous strain on the health workforce globally, especially in Southern Africa, where work overload and inadequate supplies of needed materials and equipment contribute to burnout, driving health workers out of the public health sector. Indeed, a recent study found that only 52.1% of health workers in South Africa were satisfied with their jobs and that 41.4% were actively seeking employment opportunities outside the country (14). HIV infection among health workers themselves also causes significant morbidity and mortality (15, 16). The occupational risk of TB (17, 18), including multi-drug-resistant and extremely drug-resistant mycobacteria species (19), poses further serious risk to health workers. Recognizing this, the World Health Organization (WHO), International Labour Organization (ILO), and Joint United Nations Programme on HIV/AIDS (UNAIDS) developed a guideline document to improve HIV and TB programs for health workers. This set of guidelines (20), based on a systematic review (21), was launched in November 2010, albeit specifying research gaps. The evidence included only one RCT (22) supporting workplace-based HIV and TB programs, and only a few intervention studies in healthcare workplaces (23–25), leaving decision-makers wondering whether and how to *implement* these guidelines. By 2011, very few Occupational Health Units (OHUs) in South Africa were offering HIV and TB prevention and care (26), and few, if any, were providing comprehensive programs in line with the WHO–ILO–UNAIDS guidelines. An RCT was developed, therefore, to ascertain whether OHUs in resource-challenged settings could indeed provide the comprehensive programs envisioned by these guidelines – an undertaking, we found, that was fraught with complexities. The aim of this article is, therefore, to synthesize the lessons we learned, and combine these with what others have reported in the literature, to present a list of challenges that need to be considered in preparing global population health intervention studies that would be valuable to others.

## Methods

Using the iterative framework recommended for complex RCTs (6, 7), we employed a narrative approach to describe the challenges and issues encountered as we embarked on this large collaborative RCT launched in Free State, South Africa, to evaluate whether strengthening OHUs to implement the WHO–ILO–UNAIDS guidelines *in-house* is effective, and if so, what determines successful outcomes. A narrative approach adopts qualitative methods to create meaning in order to understand the temporality and relationship between experiences. The factors that emerged were derived from data collection from a large number of discussions among stakeholders (i.e. the full research team), and refined by the authors in an iterative process. We then conducted a literature review, using the key words from our own experience. The review informed the analysis with insights from ethicists and researchers from feminist, indigenous, and post-colonial perspectives (9, 27, 28). We also included the experience reported by other research teams (5), in combination with standard guidelines for ‘intervention research’ (6). Next, we used the iterative framework developed by Campbell and colleagues (7) to situate the eight processes and lessons we felt were essential to highlight and created a checklist that might be useful to others.

## Results

The synthesized categories of considerations derived were the following and are summarized in Table 1:

**Table 1:** Factors to facilitate successful population health intervention studies in North–South Collaborations

Factor	Description	Examples/lessons from our study	Checklist of some of the challenges to consider
1) Build relationships and shared ownership	Global health research should be designed collaboratively among those with local knowledge, those with methodological expertise, and those in positions to implement findings (29). To do so requires building and maintaining trusting relationships, addressing disagreements to maximize collaborative performance. Commitment to work together, creating a common vision, frequent communication, understanding each other’s culture, and pre-planning are key determinants of successful collaborations (9, 10).	- Occupational health/infection control researchers had to build relationships with social scientists and clinicians - Researchers and practitioners of different races worked closely together - Unions had to be brought onside - Local university researchers, national and international researchers struggled to reach consensus and to establish shared ownership, creating ongoing tensions	- Varying interdisciplinary perspectives - North–South power dynamics - Racial and gender power relations - Labor–management power dynamics (especially in workplace studies) - Community -university differing priorities - Scalar differences (work unit, facility, province, nation)
2) Conduct feasibility study	Feasibility studies are small studies conducted before a main study, in order to improve the design of the main study; for example, to estimate sample size, willingness of participants, response rates to questionnaires, etc. They are useful to ascertain the priorities of the various stakeholders and attitudes toward the proposed RCT and/or its components (30, 31).	We began gathering data in 2007 in workshops and focus groups; then conducted a large baseline survey in three hospitals; then created a training program in which trainees gathered more data; we also conducted more situational analyses and further focus groups.	Researchers should continuously consider whether feasibility studies (qualitative and quantitative) would be helpful to identify stakeholder priorities and concerns, as well as to address challenges as they emerge (7).
3) Build receptor capacity	It is essential, particularly in North–South collaborations (32) that partner organizations understand not only the policies and practices that must be followed in the intervention but also the basics of the research process itself.	Our study involved several Northern students who spent many months with Southern partners; we also conducted many training sessions for local practitioners.	Considerations should include building capacity of: - local researchers and research trainees - Northern trainees (usually graduate students) - local practitioners who will be involved in operationalizing the protocols - local decision-makers
4) Create an information system to support the population health intervention	It is often necessary to create a dedicated database for large studies. However, data gathering systems for RCTs should ideally be sustainable beyond the RCT to assist in monitoring the sustainability of the intervention. Particular care to information technology issues in North–South technology transfer (33) as well as data privacy and confidentiality is needed.	We developed an information system – the Occupational Health and Safety Information System (OHASIS) – which we originally intended to have installed and used in all facilities. Technical challenges required us to fall back on paper-based forms that were faxed and data entered at the university.	-Micro considerations (do the staff entrusted with data gathering have the skills and time to do this well) - Meso considerations (does the organization support the data gathering and is providing the infrastructure – including space/computers – for this purpose) - Macro is the information technology support in place.
5) Conduct additional feasibility and pilot studies	A pilot study is a version of the main study that is run in miniature to test tools or components of the study or whether the various components can all work together (35, 36). It needs to have clearly articulated goals and procedures. It need not include randomization. Sometimes tools need to be pilot tested more than once before being used in a larger study.	- The challenges identified in the data collected in the first set of feasibility studies led to more qualitative assessments, discussions with stakeholders, and an additional pilot study, which, in turn, identified further challenges. The instruments and forms had to be re-revised many times.	- Is there a good basis to believe that the intervention will be successful (on theoretical grounds, if not previous observational studies?) If not, and new information arises during the course of the study that challenges original preconceptions, are mechanisms in place to take these into consideration?
6) Clearly articulate expectations from partner organizations, and get all stakeholders onside, with a view to scale-up from the start	A critique of population health intervention studies is that they are often not generalizable. Thinking about scale-up (37) is, therefore, needed from the start. Also, while clinical RCTs generally cover the full costs of an intervention, in population health interventions the danger of creating unsustainable processes looms even larger; engage these discussions early in the planning process.	- We were very successful in planning for scale-up, as the involvement of the Canadians began at scale (international), then proceeded to national scale, with the work at the provincial level always seen as leading to scale-up. The challenge was deciding what to fund; if the research funded all the local personnel training, it was felt that this would not be sustainable, so a balance had to be reached.	- If the study funds operational personnel to implement the intervention, will, and could, the health organization commit to maintaining such personnel should the study show the intervention to be successful? If not, have the consequences been considered?If the research funds do not fund operational personnel, and staffing levels decrease such that the study integrity is jeopardized, is there a contingency plan?
7) Develop and refine a detailed protocol	Excellent guidelines exist in his regard (6, 7); the challenge is getting to that point, and being prepared to revisit the elements of the protocol as needed.	- This has been an ongoing challenge, particularly because of different research cultures and disciplinary traditions regarding the ease and desirability of making changes along the way (which requires amendments to ethics approvals, etc.)	- Have the various partners been informed that challenges along the way may require revisiting the protocols?
8) Consider the ethical issues and obtain ethics approval	There is no algorithm to resolve conflicts among general moral considerations (31), for example, between privacy and justice, or between different conceptions of justice. The relationships built, in combination with informed institutional ethics reviews, are needed to develop the best protocol, taking the various ethical principles into account.	- Priory of ‘ethical imperatives’ differed within the team, for example, the ethical imperative to publish versus not to offend local institutions by showing problems in the system; the ethical imperative to make changes to maximize the likelihood of success of the interventions versus abiding by intended protocols.	- Has there been sufficient discussion among all parties of the benefits as well as risks from the research, not only for participants, but also at a systems level?

1. *Build relationships and encourage shared ownership of the project; nurture these relationships throughout the project and address ethical issues and differences identified*.

It is well-known that in order for projects to have a long-term impact, relationships must be nurtured and partners must develop acceptable and meaningful ways to ensure that contributions from all parties are valued (29). In our case, the partnership began at a meeting of the WHO Collaborating Centers’ Network in Occupational Health in July 2006, where the guidelines-in-development were first broadly discussed. South African representatives from the National Department of Health (DoH) and the National Institute for Occupational Health (NIOH) proposed linking Canadian and South African experts to strengthen the health of health workers in South Africa, in recognition of some of the experiences in Canada that were of particular interest to South Africans, such as those related to creating surveillance systems. We agreed to focus on workplace-based endeavors in one province, and chose Free State province after some preliminary assessments. As we wrote in our first collaborative article from this work, we chose one hospital in the Free State for the pilot work due to strong management support; a well-developed occupational health department; strong national, provincial, and academic support; and, most importantly, a Health and Safety Committee (HSC) committed to working for occupational justice that welcomed the international multi-stakeholder support (38). Support was needed not only from frontline, senior healthcare managers and provincial decision-makers but also from unions and representatives of frontline workers. Therefore, our interdisciplinary, inter-agency collaboration among Canadian team members (that began in 2004) and with South African colleagues at the national level (beginning in 2006) reached out to the several South African healthcare unions involved and Free State DoH personnel (beginning in 2007). Trust was not instantaneous, with well-described North–South power dynamics (39, 40), the legacy of racial politics (41), and questionable past ethical practices in research (42), interacting with existing tensions in labor–management power dynamics (43). Considering the importance of union support for our work, considerable effort and patience was necessary to address the multiple concerns raised by local unions. This necessitated several meetings, including bringing Canadian union leaders to South Africa to interact directly with South African counterparts (38). Another issue that arose was that while the partnership began with solid relationships among the Canadian researchers and national-level South Africa counterparts, relationships had to be built not only with healthcare workers and representatives from the DoH in the Free State where the envisioned trial would take place but also with local university colleagues, in keeping with the ethical imperative to build local capacity (44). Population health researchers would do well to heed Tuckman’s classic insight on the phases of collaboration (45) – ‘forming, storming, norming and performing’ – and recognize that delineating areas of disagreement and engaging constructive processes to address these differences, appreciating the needs of each partner, is pivotal. In our case, although the relationship began in 2008, it was not until after the pre-trial work was in its final stages (in 2013) that we fully appreciated the extent to which Canadian team members and South African researchers had different expectations and priorities within this complex multi-component intervention. Although all agreed that the ultimate purpose of the RCT was to contribute to world knowledge, build capacity, inform decision-making, and raise awareness for the need to fund such interventions, the relative importance of the various components of the intervention and how to balance the various ethical issues identified differed. Different disciplinary paradigms and research cultures heightened tensions. Scaling up nationally and internationally was important to all, but the prioritization of attention to the different scalar levels differed somewhat between the Canadians and the Free State researchers. To facilitate a process of identifying shared values and resolving differences among the collaborating teams, we engaged a university-based psychologist with expertise in conflict resolution; his involvement helped bring attention to underlying differences in disciplinary perspectives, values, and interests, showing that the disagreements should not be reduced to personality conflicts. Although collaborative research in any area requires explicit attention to issues such as authorship and shared ownership of data, this is all the more important within North–South partnerships, where a legacy of Northern researchers disrespecting Southern expertise may heighten tensions in this regard (46). Thus, nurturing relationships has to occur not only between university and community partners but also within the international research team itself.

2. *Conduct initial feasibility studies*

Campbell and colleagues (7) noted that trials of complex interventions may require iterative qualitative and quantitative data gathering to address new issues as they arise. Lancaster and colleagues presented a framework specifically for designing and conducting pilot or feasibility studies. To encourage methodological rigor during this important phase of an RCT, the authors recommend that pilot studies must include a well-defined set of aims and objectives (47). Bowen and colleagues (30), in discussing the need for evidence regarding population health interventions, indicated that feasibility studies may be indicated: 1) when community partnerships need to be strengthened; 2) when there are few previously published studies regarding the contemplated intervention technique or previous studies were not guided by in-depth research or local reality; 3) where the target population may need unique considerations; or 4) where previous interventions that employed a similar method were not successful but there is reason for optimism if changes are made. These authors provided useful advice as to how feasibility studies should be designed to answer specific questions. In keeping with their advice (30), along with advice from Campbell and colleagues (7), prior to even proceeding to pilot studies, we conducted several small feasibility studies with input from the various stakeholders to determine the feasibility of an RCT, what the essential elements ought to be, and how well-received such a study might be. The team employed questionnaires, workplace assessments, and discussion groups at one large hospital (later excluded from the trial), to obtain baseline data and input from the workforce toward designing interventions (48). Results demonstrated weaknesses in infection control knowledge and suggested the need for improved training. Inadequate supply of personal protective equipment was cited as one reason for failure to follow proper procedures; concerns about lack of training and weak management support were also mentioned. The essential role of HSCs in stigma reduction and overall improvement of occupational health and safety was also highlighted in discussion groups as necessary to include in a trial. Most importantly, however, we learned that intervention research to improve access of health workers to HIV and TB prevention and care would, indeed, be welcomed by the various stakeholders, but that there would be many pitfalls to address along the way, the most important of which are discussed further below along with how we responded to these challenges as they arose.

3. *Build capacity at all levels – including of decision-makers and practitioners*

The collaborative Canadian–South African team offered a training program at the University of the Free State (UFS) and the Free State DoH, in conjunction with national experts from NIOH and personnel from international agencies, including the WHO and ILO (48), to improve understanding of research and build capacity in occupational health and infection control. This was needed for day-to-day practice as well as for implementing the envisioned RCT. We delivered a 1-year certificate program for 28 health practitioners from across the Free State province with responsibility for HIV and TB prevention in their workplaces. Participants committed to attend three 5-day in-class sessions, as well as to conduct a relevant group project in their workplace. Significant improvements in knowledge, attitudes, and skills related to understanding research as well as practice in this area were achieved (34). Considerable challenges were also identified, not the least of which was lack of Internet or computer access, and lack of time for participating in data collection due to severe understaffing. A public sector strike that blocked access to the hospital during planned onsite training sessions also required us to improvise new training methods along the way (49). Notwithstanding the challenges involved in building receptor capacity, and indeed the ethical imperative to build local capacity to implement solutions (50), our analysis led us to conclude that such efforts are essential to the continued success of population health RCTs in LMICs.

4. *Create an information system to support the population health intervention*

Data collection at OHUs needed strengthening in terms of training and capacity, technological resources, and management support. An efficient method for capturing and analyzing the vast amount of data expected to be collected for this RCT was required. Building upon a system used in British Columbia, Canada, the Occupational Health and Safety Information System – ‘OHASIS’ was developed collaboratively by Canadians and South Africans to track occupational health indicators (33). OHASIS links to human resource databases, facilitating comparisons across occupational groups, departments, facilities, and jurisdictions while guarding confidentiality. Such a tool has facilitated considerable research in Canada (51–53) and was ideal for tracking the outcomes and related indicators needed for the envisioned RCT. The development of HIV and TB modules for OHASIS, however, was a long process and the team continues to seek the most convenient way for busy occupational health practitioners to systematically collect the data needed for operational purposes, as well as for our RCT, without adding burden. With slow Internet connectivity speeds, poor computer access, and limited computer literacy, this is an ongoing challenge. Information systems often fail (54, 55), because of a variety of factors at the micro (unit where data collection is to take place), mezzo (institutional level where information technology [IT] and other infrastructural support is needed), or macro (economic and/or political concerns beyond the level of the institution) levels. These apply equally to our experience with OHASIS (33), compounded by a high turnover rate of local IT expertise we encountered in the lead-up to our study. As we are working to bridge the well-documented ‘digital divide’ (56, 57), our hope is that by the end of the RCT, OHASIS and its local support network will be fully operational without external or researcher support. However, paper records are still needed during the RCT to mirror and support the Web-based system for collection of those indicators that must be collected at the OHU itself.

5. *Conduct additional feasibility and pilot studies*

Several additional feasibility and pilot studies were launched to address unresolved issues, such as the questions below:

 What were the barriers that we would have to address to improve OHU utilization in the trial itself?

As part of the training program described above to improve practitioner and decision-maker capacity, a study was designed by one of the trainee-groups to improve utilization of workplace HIV programs for health workers at the hospital. From January to May 2011, only 121 of the 1,900 health workers who accessed the occupational health service did so for HIV Counseling and Testing (HCT). In contrast, 568 health workers came for Hepatitis B immunization. This study suggested that health workers at this facility could be under-utilising the OHU due to fear of being stigmatized, fear of breach of confidentiality, lack of knowledge of the HIV program, misconceptions and attitudes toward the OHU service and HIV program, and/or a misperception of policies and their implementation. To improve utilization, the group responsible for this project recommended more education and training for frontline workers and OHU staff, improved promotion to increase awareness about the OHU and greater emphasis on ethical principles such as confidentiality (58). A Canadian Master’s student working within the research program also explored this issue, using focus groups as well as survey analysis, and derived similar conclusions (59). Since November 2011, the OHU at this facility (excluded from the RCT) has been offering a comprehensive HIV program where HCT, routine monitoring of CD4 counts, drug readiness and access to treatment is offered. The experience in this OHU will continue to provide valuable information to complement the RCT across the other 27 OHUs in the province’s healthcare system that were randomized for the trial.

How should TB screening and follow-up occur?

Two small projects addressed this question. A second group in the Free State training program conducted meetings with stakeholders to explore ways to strengthen the OHU for addressing TB. This study also concluded that confidentiality was a major concern (60) and that unit managers should not be involved in the TB screening process. Another Canadian Master’s student also sought to determine the best way to offer TB screening. A best–worst scaling choice experiment was incorporated into the protocol to quantify attributes that may influence a healthcare worker’s decision to be screened for TB. This study found that cost and wait time had the highest relative preferences, with administration of testing at the occupational health clinic indeed being the preference; interestingly, though, this was more strongly expressed by blacks, colored, and South Asian than by white healthcare workers (61).

How receptive will the workforce be to questionnaire surveys at the workplace?

Our team also conducted a large workforce baseline study across three large public hospitals in the Free State, asking about uptake of TB screening, vaccination and health and safety practices, as well as views on confidentiality, stigma, and a variety of issues related to occupational health and infection control. In addition to the interesting findings of the survey itself, it demonstrated that workforce surveys can indeed be successfully implemented with a high response rate. However, in conducting this survey we ascertained some reluctance in the workforce for longitudinal follow-up – in other words, individuals, despite willingness to participate in anonymous cross-sectional surveys, were not sure they wanted to be contacted again. This created a dilemma for the research team as to whether to proceed with sequential non-linked cross-sectional surveys, invest more funds for much larger sample sizes to account for a predictably high loss to follow-up in randomized longitudinal analysis, or proceed with the intervention components as planned, with more heavy reliance on qualitative outcome.

Do we have robust instruments to measure outcome?

Finally, we extensively pilot tested our instruments. The OHU data collection tool was revised several times before launching. In addition, we developed a workplace service provision tool to assess the current status and capacity of occupational health services, and a tool to measure stigma in the workforce related to HIV infection in healthcare workers, as such an instrument had never previously been developed. We note that Daivadanam and colleagues (5), in their study of dietary behavior in Kerala, pilot tested their instruments three times before launching their cluster RCT. Importantly, the challenge of questionnaire administration in LMICs with an understandably strong history of mistrust makes the issue of how to collect reliable and valid data one that can easily overshadow issues of face and content validity of the questionnaire itself.

6. *Be clear as to what activities are to be ‘research funded’ and what is expected as ‘ongoing program funding’*

Corbin and colleagues describe their experience working with an organization called ‘KIWAKKUKI’ and suggest that if scale-up is done correctly, partnerships and programs can create synergy and pave the road for further growth. For this to happen, responsibilities related to funding and capacity building must be clearly outlined from the beginning (62). The studies and interventions described above not only informed the RCT but also raised awareness of OHS issues at the Free State DoH and contributed to the receptivity of the Free State’s health system to workplace HIV-TB interventions. Results of the studies were presented at a research day attended by all stakeholders, including senior representatives from the hospitals (including chief executive officers [CEOs]), provincial health department executives, local unions, and officials from the national level (63) as well as at a feedback session for the unions. Similarly, special visits were made to each facility to discuss findings with hospital management. These presentations created an opportunity for open dialogue regarding the way forward and for obtaining further support for the RCT.

In preparation for this RCT, a review was conducted of the 27 OHUs in public health facilities in the Free State. This review included information on staffing and training received by staff, available medical supplies, privacy and confidentiality, and services rendered at OHUs. The review was undertaken by the clinician (KU) supporting the RCT during visits to the OHUs as well as through the use of self-administered questionnaires that were completed by the OHU nurses during a provincial occupational health meeting. Problems that were identified by the review included: Not all OHUs were staffed by full-time nurses or had access to doctors; training on the management of TB and HIV was lacking; there was a shortage of basic medical equipment at some clinics; and at some of the OHUs, the design of some consulting rooms did not support privacy and some consulting rooms were located some distance from clinical support services.

In the lead-up to the RCT, we attempted to address these issues by engaging with hospital management, including the CEOs as well as providing training. As a result of these interventions, by mid-2013 we had achieved the following positive changes: two hospitals had appointed an extra full-time nurse to staff the OHU; almost all occupational health nurses had been trained on TB and HIV management; access to basic medical equipment such as blood pressure machines and hemoglobin meters had improved at some OHUs; and two OHUs had moved to new locations to ensure more privacy and confidentiality for health workers accessing the service as well as closer proximity to clinical support services.

In a series of further meetings and presentations, stakeholders offered input, endorsed the RCT, and were encouraged to continue to provide input at all stages of the planning and implementation process. As discussed below, the team grappled with policies within the health department that would have made it difficult to hire additional staffing for the various components of the intervention. It was, therefore, agreed by all that funding from the research grant could support the training, monitoring, and evaluation of outcomes but not additional operational staff, because the intervention would otherwise not be sustainable, even if shown to be effective. Global economic factors and other policy considerations continuously impact public sector funding, especially in LMICs (64, 65), and must always be taken into account as possible factors that could undermine success of such trials.

7. *Develop and refine a detailed protocol*

Following the steps above, we then developed an RCT protocol, explicitly aiming to determine how a comprehensive approach to strengthening TB prevention and follow-up in the workplace, expanding testing and treatment at OHUs to include the offering of anti-retroviral therapy onsite, together with improving service awareness, confidentiality provisions, and stigma reduction efforts produces impacts on testing, treatment, sick time, and death rates among workers in public-sector hospitals in the Free State. OHUs at all 27 hospitals in the province that had OH nurses appointed (with the exception of the pilot hospital) were paired (there was one group of three) and then one of each pair randomized to either the intervention or control arms. Agreement was obtained from the DoH to ensure that all health workers would be trained in TB management, as well as the diagnosis and management of TB, HIV/AIDS, and sexually transmitted infections in primary care settings and TB infection control (use of natural ventilation, encouraging cough etiquette, appropriate use of personal protective equipment, early identification of suspect cases, segregation/social distancing and fast tracking of patients at risk for communicable respiratory infections, etc.) and to provide adequate supplies (such as N95 respirators). However, the issue arose as to whether the training and follow-up provided by the department was sufficient to call this a model intervention for purposes of the trial. In clinical interventions, it is customary for the research project to fund the full clinical costs; however, for this intervention, as noted above, some team members raised concerns as to whether the program would be sustainable after the study if the expenses of training, monitoring, and follow-up were borne by the study. It was eventually decided that the study would indeed support (monitor and follow-up) the efforts in the 14 intervention sites but not the 13 comparison sites. It was further agreed that the research team would monitor these costs and communicate these in a final report to the DoH.

8. *Consider the ethical issues and obtain ethics approval*

**Fig. 1:**
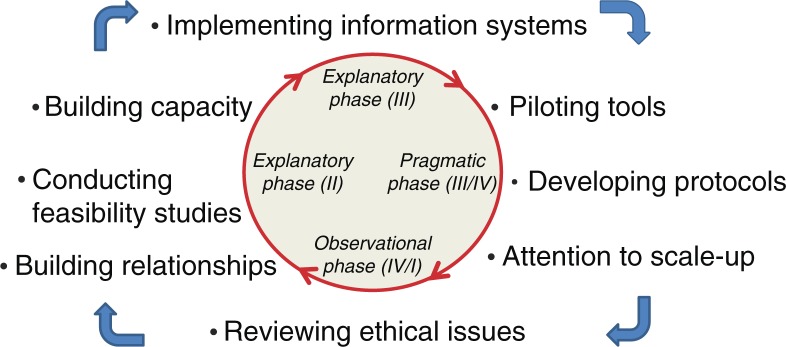
Iterative considerations in launching complex population health cluster-randomized controlled trials. Note: Adapting Campbell et al. (7)’s four-phase iterative approach to complex RCT.

As suggested by Schuftan (66), all health professionals and researchers have political, ethical, and ideological motivations that influence their actions in the realm of global health. Acknowledging these inherent biases and subsequent political, social, and systematic implications is a critical part of the research process. Many discussions occurred throughout the above process related to the dual goals of maximizing both the health of the collective (the workforce, patients, and the community at large) as well as respecting the privacy rights of workers. Methods were derived to carefully guard confidentiality, and the support of the unions was considered key in moving forward. Ethics approvals were obtained at the Canadian institution; University of British Columbia (UBC), and South African institution, UFS. Specifically, four different Research Ethics Board certificates were obtained at UBC to cover the various data collection exercises noted above; two were obtained at UFS (the first set of activities occurred before UBC engaged UFS as partners; and a separate ethics approval process was obtained for the various workplace field studies conducted by the trainees in the research and training program). More importantly though, we endeavored to implement the principles of solidarity and social justice that we believe are paramount to the ethical conduct of global health research (50). Further, as shown in Fig. 1, the consideration of ethical issues must begin at the very beginning of the collaboration, and be seen as very much an iterative process, continuously informed by the qualitative and quantitative data that emerge during all phases of the research.

## Discussion

It is interesting to consider the contrasting implementation and evaluation processes in clinical RCTs and population health interventions. Clinical RCTs use a more reductionist, deterministic approach where the protocol is published and remains fixed. Results and learning are shared once the study is complete, as sharing results too soon can compromise the ability to publish. In contrast, population health interventions develop more organically; there are interacting components and different outcomes. The phased process allows flexibility and ability to respond to unanticipated challenges such as a strike and tailoring of the intervention as circumstances require.

As noted by others (67), effective public health programs require change at many levels and require an understanding of the contexts. Although we agree with Victora and colleagues in their article entitled ‘Beyond RCTs’ that it is necessary to develop evaluation standards and protocols for use when RCTs are not appropriate (68), we believe that strong RCTs can be developed to evaluate complex population health interventions in LMICs, if proper attention is paid to ensuring that necessary conditions are satisfied. Campbell and colleagues argued for conceptualizing complex interventions in phases iteratively informed by both qualitative and quantitative data (7); this article builds on their approach by specifying challenges that must be addressed in global population health interventions, creating a checklist to highlight tough issues that need to be confronted. This notably includes developing and maintaining trusting relationships, as noted by others (8, 32), recognizing that a process of critically engaging differences of opinion will likely be needed. Success also includes paying attention to scale-up and sustainability issues from the very beginning; without the vision of how the findings can be used, the stakeholder support needed not only to implement findings but also to assist in operationalizing the research itself, may well be undermined.

Because population health interventions are complex, especially in LMICs, feasibility and pilot studies are warranted to ensure that the complexities are properly understood; given the power relations inherent in global health research, such efforts are even more warranted. Building capacity, as noted by Benatar and Singer (31), is also essential, particularly in global health research, where the capacity to undertake the basics of intervention research is often quite limited and fostering local expertise is an ethical imperative (50). Similarly, population health interventions are often conducted in settings where data collection procedures are weak, therefore, addressing the challenges of creating robust information systems must also be considered. Of particular importance is paying attention to the individual versus collective ethical tensions (27).

The WHO–ILO–UNAIDS guidelines (20) and associated systematic review (21) called for better intervention research to improve access for health workers to prevention and care services for HIV and TB. We believe that we have developed a robust RCT protocol to address the identified gaps. The impact of these efforts will be best evaluated in several years following the completion of the RCT.

The considerations and challenges outlined are not meant to be sequential, but rather frame a checklist, to be refined as additional experiences in meeting the challenges of global population health intervention research are documented, exchanged, and critically evaluated. Our team was able to obtain various small grants along the way to building this RCT; researchers should likewise consider seeking such pre-trial grants to strengthen their eventual efforts. Although obtaining funding for pilot studies and capacity-building programs may not always be feasible, such efforts definitely merit consideration.

Finally, this article has its limitations. The analysis was conducted *post hoc*, retrospectively reflecting upon what had been learned, rather than prospectively following a framework for analysis at each step. This relates to a second limitation; although some articles were drafted (and a few published) along the way, much of the data gathered in the preliminary steps, feasibility studies, and pilots were never actually published. Thus, the lessons learned were not put to peer-review for input that could have informed next steps and better shaped the insights in this article. Nonetheless, the use of the Campbell et al. framework, in conjunction with the results of a literature review, helped give shape to the reflections reported.

## Conclusions

Just as in clinical trials, there are preconditions that must be met before launching a definitive RCT; so, too, for population health research, especially in North–South partnerships. The challenges can be complex but should not be ignored. Otherwise there is great danger that high quality evidence will be biased to contexts that favor implementation of interventions of narrow scope that are easier to evaluate – and that pressing global health concerns will continue to be under-researched. Albert Einstein observed that ‘everything that can be counted does not necessarily count; everything that counts cannot necessarily be counted’. The challenge for global population health researchers is to carefully analyze the complexities that make the ‘counting’ difficult, and creatively establish iterative evaluation methods that meet the realities in these challenging settings.

RCTs of multi-component population health interventions require considerable planning and investment of time and resources, but can, indeed, be successfully implemented, even, as shown in our study, in addressing such sensitive issues as prevention and treatment of HIV and TB in health workers. The eight considerations, and related checklist articulated here can be used as a guiding framework, with the hope that greater attention to the processes that are involved in such endeavors will lead to improved timeliness and quality of global population health intervention research.

## Highlights

1. Time-consuming challenges exist in preparing complex population health RCTs, especially in North–South collaborations.
2. Careful consideration of the needs for relationship building, feasibility and pilot studies, information systems, capacity building, stakeholder support, and attention to scale-up and ethical concerns can lead to strong RCT protocols.
3. RCTs to enhance implementation research (even in difficult areas such as in workplace HIV and TB prevention and care) are feasible.
